# Supplementary figures and images for: Local-scale phylodynamics reveal differential community impact of SARS-CoV-2 in a metropolitan US county
Source: PLoS Pathog. 2024 Mar 26;20(3):e1012117. doi: 10.1371/journal.ppat.1012117 (PMC10997136; doi:10.1371/journal.ppat.1012117)

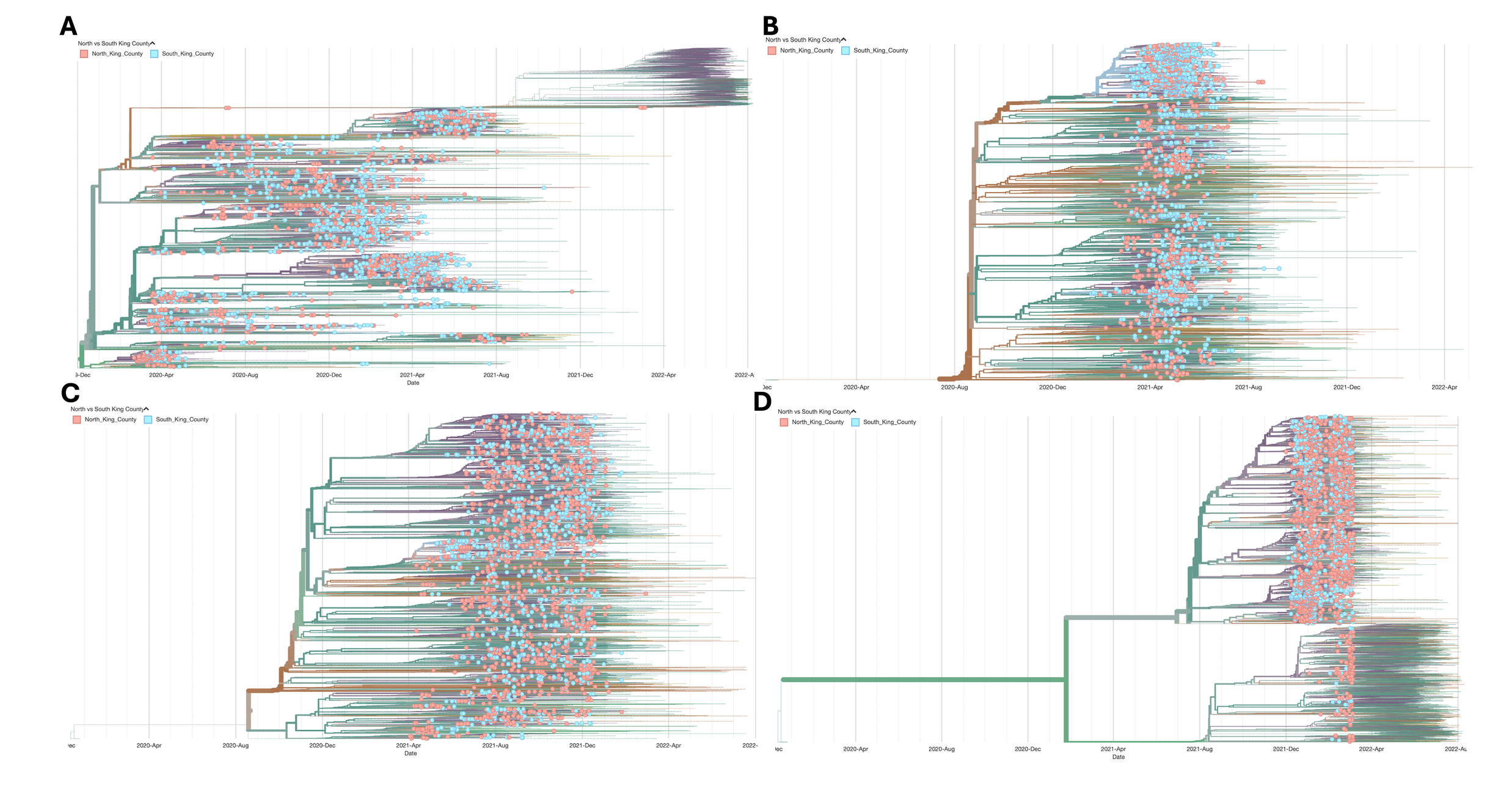

Supplement: S1 Fig — Trees are filtered to highlight genomes from King County among contextual sequences from around the globe. Tip color represents the region within King County, with pink corresponding to North King County and blue representing South King County. Branches are colored based on inferred ancestry. Panel A represents all variant clades excluding Alpha, Delta, and Omicron (the full tree can be explored interactively at https://nextstrain.org/groups/blab/ncov-king-county/other), the other panels represent Alpha (B, https://nextstrain.org/groups/blab/ncov-king-county/alpha), Delta (C, https://nextstrain.org/groups/blab/ncov-king-county/delta), and Omicron (D, https://nextstrain.org/groups/blab/ncov-king-county/omicron. (TIF) [file ppat.1012117.s001.tif]

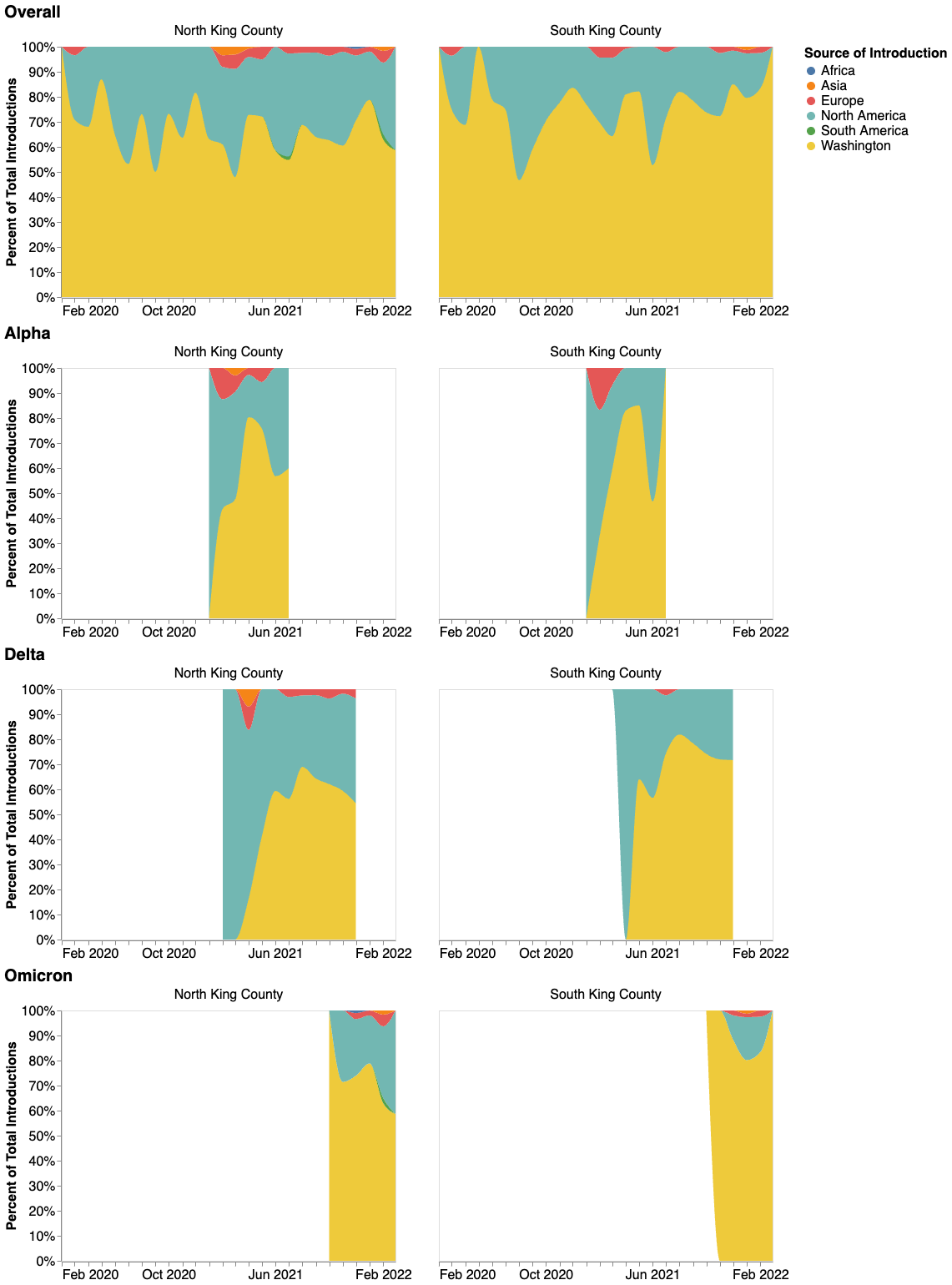

Supplement: S2 Fig — The left column is introductions into North King County, the right into South King County. The panels show how the inferred geographical source of each introduction changes over time as a percentage of all introductions into the regions for that time period. The top row contains all the introductions among the four different time-resolved phylogenies. Each subsequent row represents a different variant studied and is labeled accordingly. (TIF) [file ppat.1012117.s002.tif]

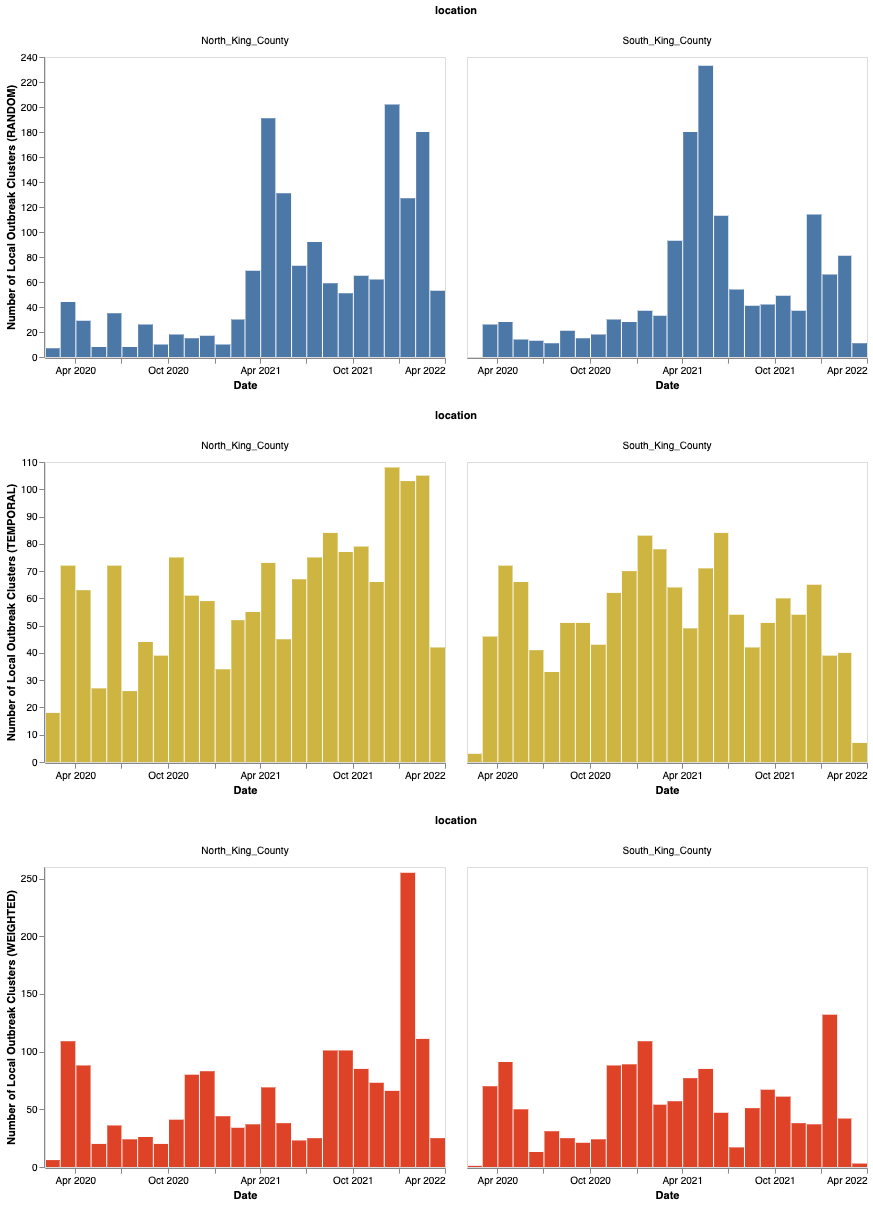

Supplement: S3 Fig — Number of local outbreak clusters over time by subsampling scheme: random (A, Blue), equal temporal weighting by year-week (B, Gold), and subsampling weighted by daily hospitalizations calculated using a 14 day moving average (C, Red). (TIF) [file ppat.1012117.s003.tif]

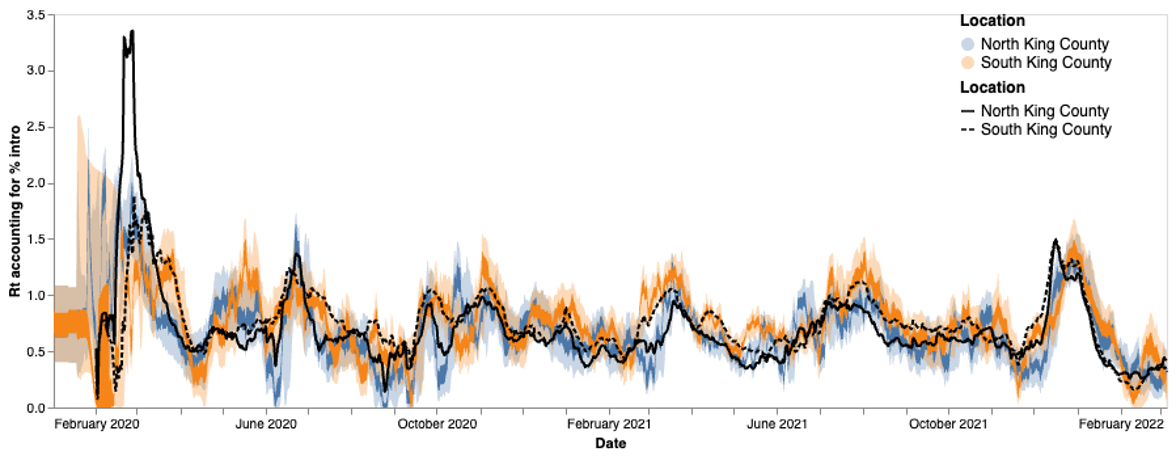

Supplement: S4 Fig — (TIF) [file ppat.1012117.s004.tif]

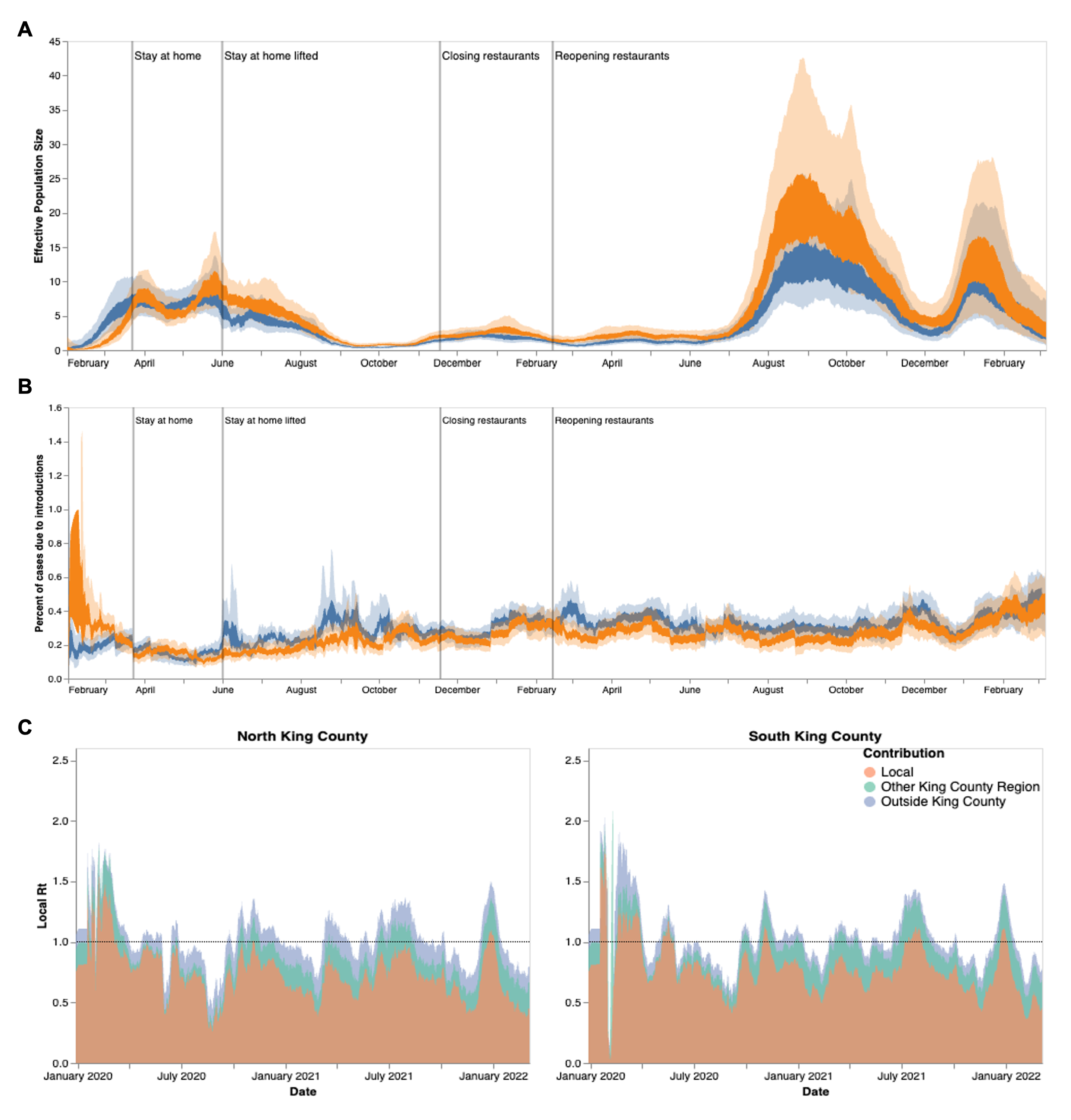

Supplement: S5 Fig — Results presented above were inferred using 3000 sequences subsampled using equal temporal weighting by year-week. Analyses presented, as defined previously, are: effective population size over time (A), percent of cases due to introductions (B), and local Rt estimations divided by region and source of contribution (C). Orange denotes South King County; blue denotes North King County. (TIF) [file ppat.1012117.s005.tif]

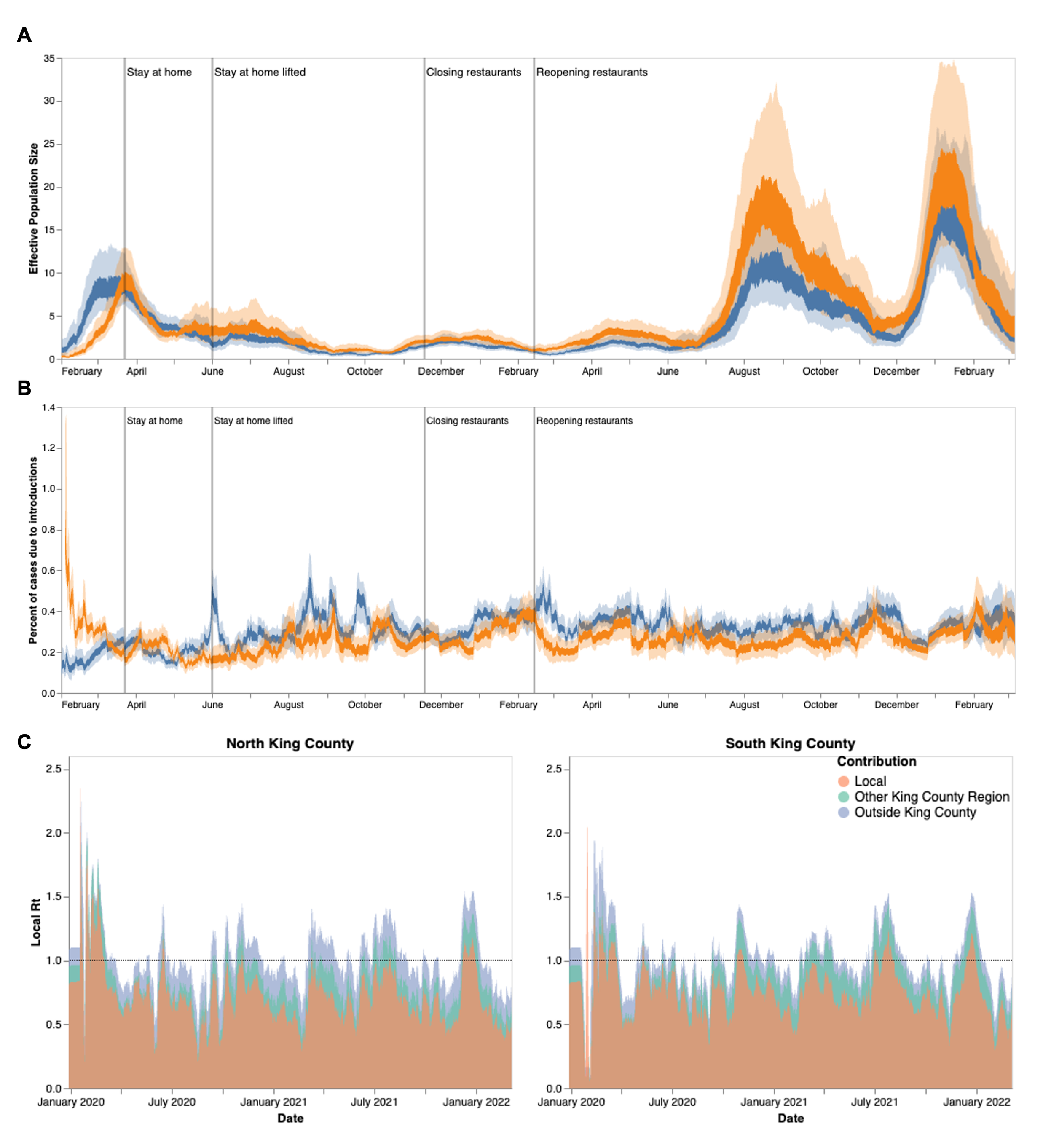

Supplement: S6 Fig — Results presented above were inferred using 3000 sequences subsampled using weighting by hospitalizations over time using a 14 day rolling average. Analyses presented, as defined previously, are: effective population size over time (A), percent of cases due to introductions (B), and local Rt estimations divided by region and source of contribution (C). Orange denotes South King County; blue denotes North King County. (TIF) [file ppat.1012117.s006.tif]
