# Supplementary material for: Local-scale phylodynamics reveal differential community impact of SARS-CoV-2 in a metropolitan US county
Source: PLoS Pathog. 2024 Mar 26;20(3):e1012117. doi: 10.1371/journal.ppat.1012117 (PMC10997136; doi:10.1371/journal.ppat.1012117)
Supplement: Attachment — Submitted filename: ncov_kc_plos_path_response_and_cover_letter.pdf [file ppat.1012117.s009.pdf]

Dear Dr. Stern,

Thank you for your support and continued interest in our paper “Local-Scale phylodynamics reveal differential community impact of SARS-CoV-2 in metropolitan US county.” This is the resubmission of manuscript PPATHOGENS-D-23-01656.

Attached you will find our point-by-point response letter. Editorial and reviewer comments are shown in black, and our responses in blue.

The majority of the revisions have been textual in nature surrounding clarification of the data, limitations of our work, and further contextualization. At the suggestion of the reviewers, we have included two new supplemental figures to highlight the viral genetic diversity of SARS-CoV-2 in King County, WA via our time-resolved phylogenies (Supp. Figure 1) and to investigate how the source of introductions into King County change over time, by variant, and by sub-county region (Supp. Figure 2). We also rearranged the order of the main figures so that original Figure 6, which contains important contextual information about the socioeconomic inequities in King County, is now Figure 1 embedded within the Introduction rather than in the Discussion section. We have also attempted to make our work more applicable to a broader audience by expanding the final paragraph in the discussion section to describe how our local scale genomic epidemiology approach could help inform public health action in areas of epidemic and socioeconomic differences beyond just King County.

Again, thank you very much for your consideration.

Yours sincerely,

Miguel Paredes, Nicola Müller, and Trevor Bedford, on behalf of all co-authors

—---

Both reviewers agree this is an excellent high quality paper and have a series of important small suggestions. Of note esp. is comment 6 of reviewer 2 - it is important to contextualize this paper for broad readership, and give examples of how this study can inform us beyond the particular case of north/south king county (which may very well reflect similarity to many other large cities?).

Reviewer Comments (if any, and for reference):

Reviewer's Responses to Questions

Part I - Summary
Please use this section to discuss strengths/weaknesses of study, novelty/significance, general execution and scholarship.

Reviewer #1: The study investigates the transmission dynamics of SARS-CoV-2 at a local level in King County, Washington, utilizing genomic, epidemiological, and mobility data from February 2020 to March 2022. The authors employ an advanced analysis method to uncover significant disparities in transmission patterns between North and South King County following the implementation of stay-at-home orders. Notably, South King County experienced higher reported cases, hospitalizations, and sustained local transmission compared to North King County, where new cases are primarily influenced by importation from outside the county. These disparities are attributed to diverse mobility and socioeconomic challenges faced by residents of South King County, emphasizing the importance of considering local conditions in pandemic management. The study is exceptionally well-written report, the results are well presented, and includes an impressive phylodynamic analysis pipeline. A few remarks are listed below for consideration.

Reviewer #2: This study uses 11,737 SARS-Cov-2 sequences sampled from January 2020 – March 2022 from people living in King County, Washington (which includes the city of Seattle) to study patterns of virus transmission, including community spread versus the role of outside introductions in driving the epidemic. These approaches have been successfully used around the world to evaluate the impact of non-pharmaceutical interventions on controlling virus transmission.

We thank both reviewers for their enthusiastic support and their thoughtful comments that made the manuscript stronger.

Part II – Major Issues: Key Experiments Required for Acceptance
Please use this section to detail the key new experiments or modifications of existing experiments that should be absolutely required to validate study conclusions.

Generally, there should be no more than 3 such required experiments or major modifications for

a "Major Revision" recommendation. If more than 3 experiments are necessary to validate the study conclusions, then you are encouraged to recommend "Reject".

Reviewer #1: - In the Methods section, please provide a comprehensive explanation of the selection process for contextual sequences, as identifying local clusters through phylogenetic inference is a critical aspect of this study.

We agree that further explanation of the selection process for contextual sequences is needed and we have updated the Methods section under the subheading *Clustering* as follows:

> *Contextual sequences are needed in order to investigate how King County samples relate to regional and global viral diversity, and to identify local outbreak clusters specific to King County. Given that cluster identification is conditional on the number of background sequences that interdigitate large clades on the phylogeny, we attempted to maximize the number of contextual sequences within the bounds of reasonable computational efficiency. We prioritized sequences from WA and North America in order to optimize regional context. For each variant, we specified contextual data sampling to include up to 10,000 genomes per time-period from WA, sampled from all counties and months, up to 7000 genomes per month from other US states, and up to 5000 genomes per month from the rest of the world. In each variant specific phylogeny, contextual sequences comprise 83-86% of the total number of sequences. While we expect the number of the clusters to increase with an increasing number of contextual sequences, prior work has shown that changes in the proportion of background sequences that make up the analytical dataset above a proportion of 50% have limited impact on the number of clusters identified and mean cluster size (Fig. S13 in (2)), and downstream phylodynamic analyses.*

The referenced figure (Fig. S13 in (2)) refers to a phylodynamic study in Washington conducted by co-authors of the current manuscript where the authors investigated the impact of downsampling the proportion of background sequences in their sample on the number of clusters identified and the mean cluster size using the same parsimony algorithm used in the present study. The supplemental analysis, reproduced below, shows limited impact of increasing background sequences above a proportion of ~50%, lending strong support for the robustness of our results.

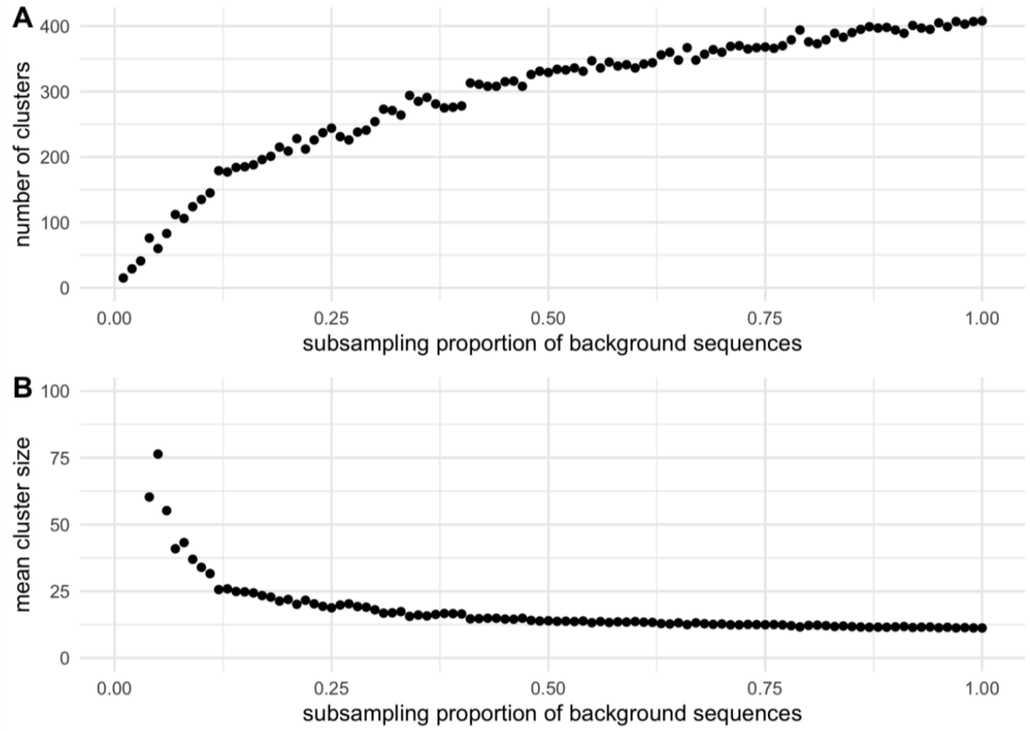

Fig. S13. Dependence of the local outbreak clusters and the number of background sequences used. Here, we estimated the number (**A**) of mean size (**B**) of local outbreak clusters depending on the number of background sequences used during the clustering. To do so, we randomly subsampled different proportions of the background sequences (x-axis) and repeat the clustering. We then computed the number of clusters and the average sizes of cluster(y-axis) depending on the proportion of background samples used relative to the full dataset.

Reproduced from Müller NF, Wagner C, Frazar CD, Roychoudhury P, Lee J, Moncla LH, et al. Viral genomes reveal patterns of the SARS-CoV-2 outbreak in Washington State. Science Translational Medicine. 2021 May 26;13(595):eabf0202.

Reviewer #2: 1. This paper discusses the role of outside introductions. Did North King county and South King county appear to have the same geographical sources for these introductions? Did the geographical source of outside introductions shift over the course of the pandemic?

We agree that this is worth exploring further. As such, we have used our four large variant-specific maximum likelihood (ML) trees to investigate the geographical sources of introductions into King County. To do so, we inferred the ancestral region for each node on the ML tree and then extracted out the subtrees whenever there was a migration event from a non-King County region into King County. The results of this analysis is presented below:

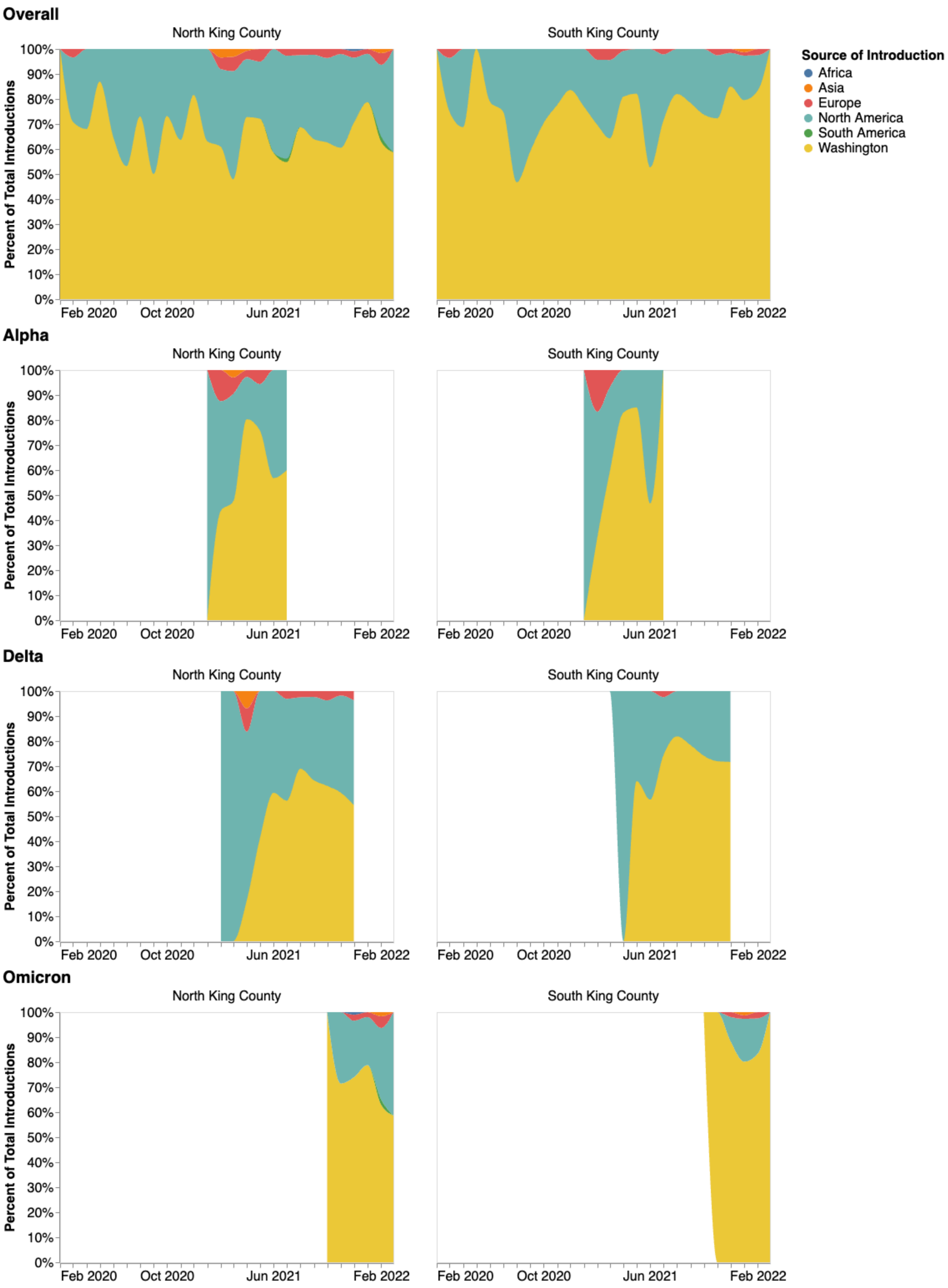
Overall
Alpha
Delta
Omicron
North King County
South King County
Percent of Total Introductions
100%
90%
80%
70%
60%
50%
40%
30%
20%
10%
0%
Feb 2020
Oct 2020
Jun 2021
Feb 2022
Source of Introduction
Africa
Asia
Europe
North America
South America
Washington

Supplementary Figure 2: Source of introduction for each identified King County cluster. The left column is introductions into North King County, the right into South King County. The panels show how the inferred geographical source of each introduction changes over time as a percentage of all introductions into the regions for that time period. The top row combines all the introductions among the four different time-resolved phylogenies. Each subsequent row represents a different variant studied and is labeled accordingly.

In the figure above, we can see that Alpha and Delta arrived first into King County from outside the state before arriving or spreading into the larger Washington region. Additionally, we see Alpha arriving into King County from Europe as well, which supports the validity of our results since Alpha first arose in the UK. We see that as time progresses, the source of introductions switches from mainly North America (excluding WA) to predominantly from within Washington. Omicron, conversely, seemed to spread into King County mostly from Washington State. Additionally, we see that North King County has a larger proportion of viral introductions coming outside Washington while the majority of introductions into South King County come from within the state.

We have added in the description into the results section:

> *We also analyzed the inferred ancestral location for all clusters over time divided out by the dominant variant waves (Supp. Fig 2). We found that Alpha and Delta arrived first into King County mainly from other US states before spreading into the larger WA region, with Alpha also arriving from the UK where it originated. As time progressed, the source of introductions switched from mainly North America (excluding WA) to predominantly from within Washington (excluding Omicron which was introduced into King County primarily from WA). Additionally, we saw that North King County has a larger proportion of viral introductions coming from outside WA while the majority of introductions into South King County come from within the state.*

2. One question during the pandemic was why alpha did not take off in the US the way it did in the UK where it emerged. When was the variant introduced into King County? Did you observe lots of introductions but not much community transmission? Any idea why?

> Look at mean cluster size over time (per month) by variant like in figure 2.

Using a US-focused time-resolved phylogeny found on nextstrain.org (https://nextstrain.org/ncov/gisaid/north-america/all-time), we can see that Alpha made up more than 30% percent of sequenced cases in the first half of 2021. A Washington focused phylogeny maintained by the Washington State Department of Health (https://nextstrain.org/groups/waphl/ncov/wa/all) shows similar results with Alpha making up over 50% of included cases in May 2021, although we see concurrent competition with

co-circulating variants such as Epsilon, Gamma, and then a rapid dominance by Delta by July 2021.

Previous work done by some of our co-authors, have looked into quantifying the contribution of each variant on the SARS-CoV-2 epidemic in WA State (preprint found here: https://www.medrxiv.org/content/10.1101/2021.12.09.21267544v2) . Looking at Figure 1B (reproduced below), we see that Alpha drove an epidemic wave in Washington that would not have otherwise occurred. In Fig 1C, we can see the high frequency of Alpha in WA at the time but also appreciate the co-circulation of other variants and the eventual dominance of Delta.

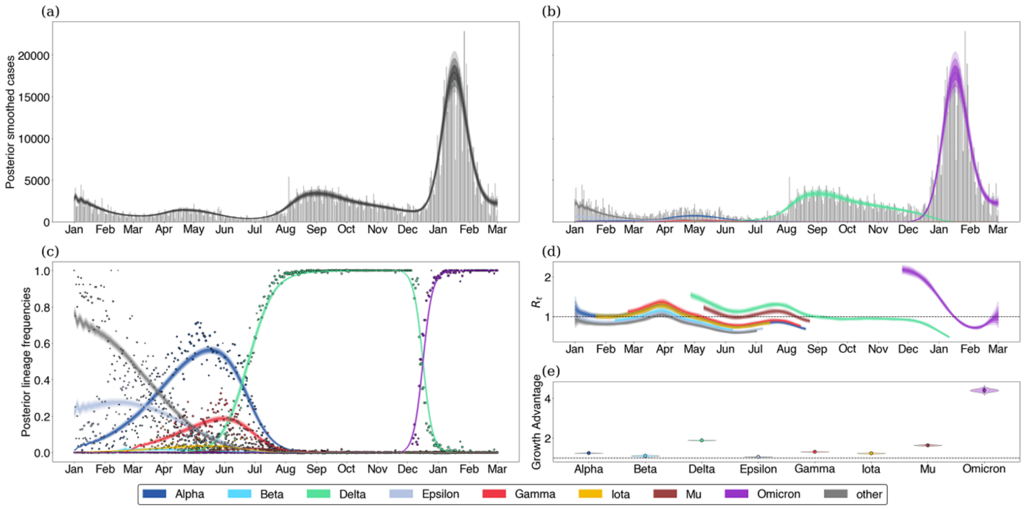

Figure 1. Fitting the fixed growth advantage model to Washington state data. (a) Posterior expected cases without weekly seasonality in reporting rate. Gray bars are observed daily case counts, while blue lines are model inferences with 50%, 80% and 95% credible intervals. (b) Posterior expected cases by variant. Each colored line is a different variant with intervals of varying opacity showing 50%, 80% and 95% credible intervals. (c) Posterior variant frequency against observed sample frequency. Dots represent observed weekly frequencies in sequence data and each colored line is a different variant with shaded CIs. (d) Variant-specific effective reproduction numbers. (e) Posterior growth advantage by variant.

When looking specifically at King County, we can see an increase in both cases and hospitalizations between April-July 2021 in Figure 2C-D reproduced below, during which Alpha had a variant frequency greater than 30% in Washington State. This increase in both cases and hospitalizations suggests that Alpha did have a significant role in King County (alongside co-circulating variants such as Epsilon and Gamma) but was subsequently outcompeted by Delta, which represents the wave of cases that follows Alpha.

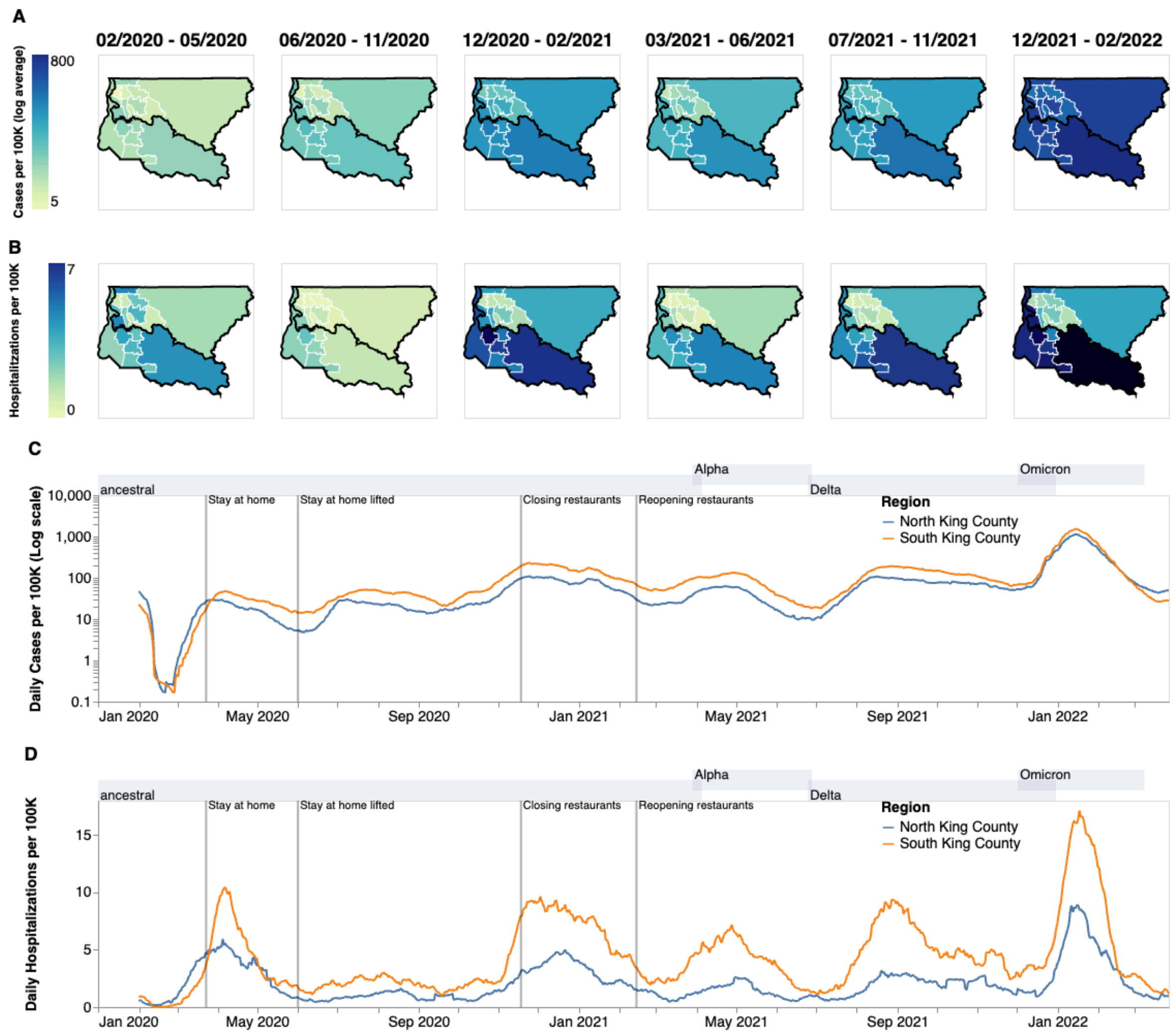

Looking at Figure 6 (reproduced below), we don't see an obvious change in the percent of cases due to introductions for either region during the period of high Alpha frequency when compared to the previous months.

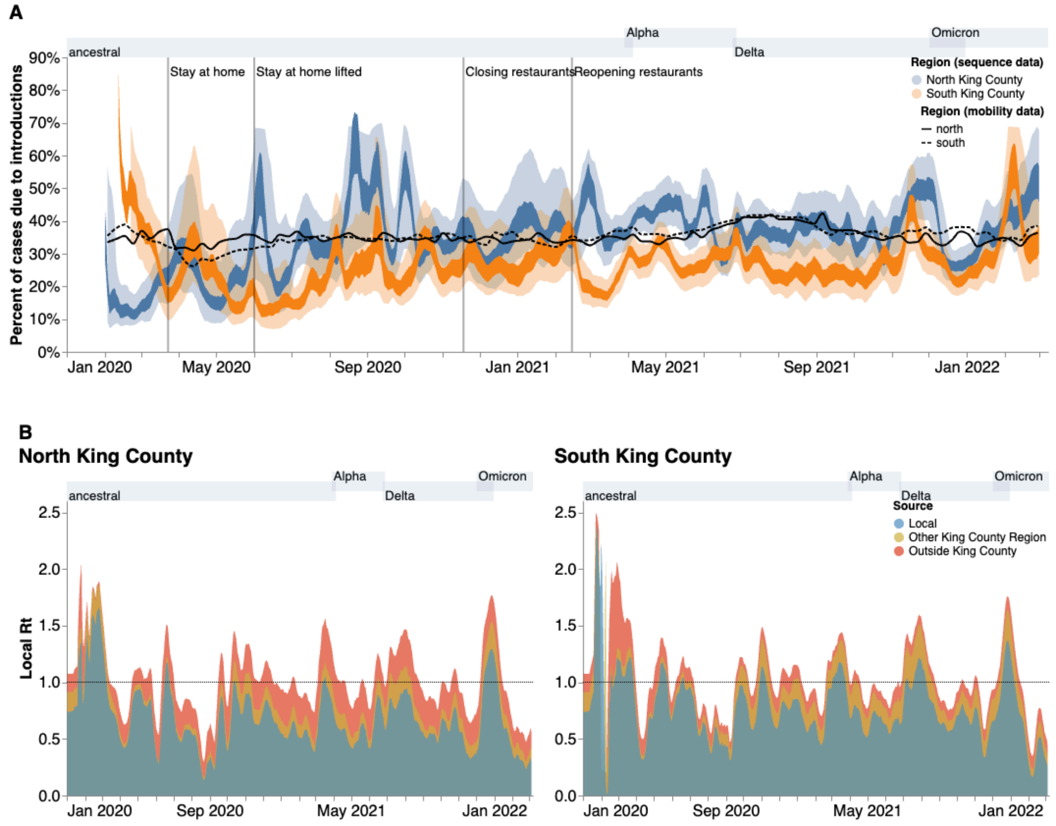

With regards to when Alpha was introduced into King County, we can look at the Supp Fig. 1, which was included in the response to question #1 above, and see that the earliest introduction we find in our sample is January 2021, concordant with when the first case was found in WA state (https://newsroom.uw.edu/news-releases/lab-detects-washingtons-1st-cases-uk-covid-19-strain)

3. Is there any parameter that is a good predictor of whether a transmission chain persists for a long period? Seasonality? Population immunity? Location of origin? Timing related to non-pharmaceutical interventions? Or does it appear to be stochastic?

We agree with the reviewer that this is an important question. The use of MASCOT-GLM in our study allows us to not only inform our estimates of effective population size (Ne) and migration rates, but also to quantify the effect size of the contribution of each predictor (new Fig. 4 below).

We find that hospitalizations one week in the future is the predictor with the strongest effect for informing effective population size, which is related to larger transmission chains.

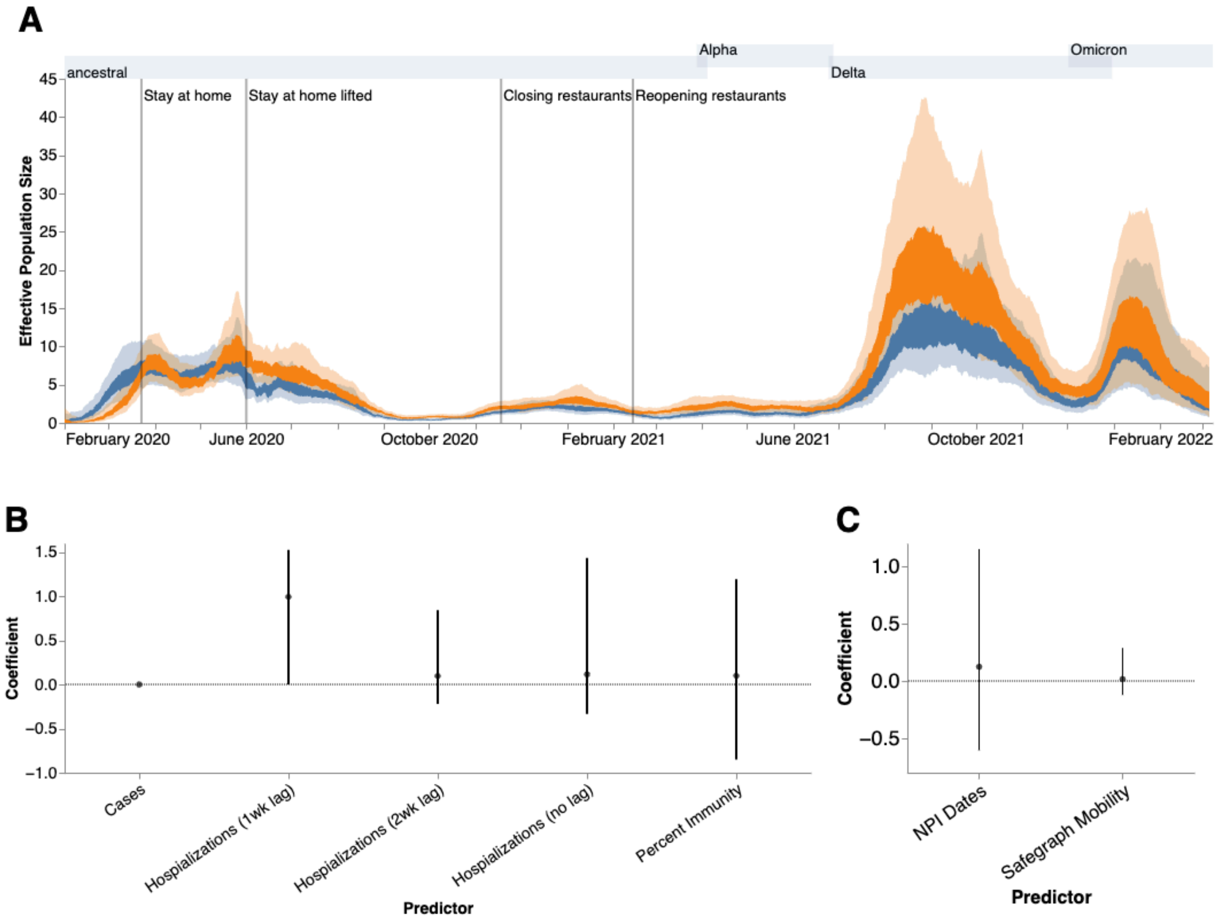

Figure 4: Phylodynamic Analysis via MASCOT-GLM. (A) Estimates of effective population sizes from Feb 2020 to March 2022 in North (blue) and South (orange) King County using 3000 randomly subsampled sequences. The inner band denotes the 50% highest posterior density (HPD) interval and the outer band denotes the 95% HPD interval. Vertical gray lines denote dates of non-pharmaceutical interventions in Washington State. Gray shaded regions denote alpha and omicron variant waves; alternative non-shaded regions represent non-variant and Delta waves, respectively. **(B)** Estimates of model predictor coefficients for *Ne* estimation and **(C)** for migration rate estimation. All of the predictors displayed on the x-axis were included in the analytic model. Dark line represents median estimates, light bands represent 95% HPD. Gray shaded regions above each figure show the time periods during which ancestral virus, Alpha, Delta, and Omicron respectively represented greater than 30% of sequenced case

We also included the following information in the Results section (new information in *italics)*:

> We then employed phylodynamic inference methods on the identified outbreak clusters to analyze SARS-CoV-2 spread in the county. Following subsampling, we used a

MASCOT-GLM approach with relevant predictors on a random subsample of 3000 sequences from our dataset of local outbreak clusters to reconstruct SARS-CoV-2 transmission dynamics (Supp. Fig. 2). Phylodynamic estimates of the effective population size (N_e) of the virus in both King County regions over time mirror patterns seen in both confirmed COVID-19 hospitalizations and cases: while the *Ne* in North King County is initially greater until the end of March 2020, following WA stay-at-home orders, we find a consistently greater *Ne* in South King County throughout the study period (Fig. 4A). *We also find that hospitalizations one week in the future was the most informative predictor for effective population size in our model (Fig 4B), while the migration rate predictors were not significantly informative (Fig 4C).*

We believe, however, that robustly answering the specific question of what are the best predictors to whether or not a transmission chain persists for a long period is outside of the scope of this study as it would require a different study design, methodology, and data sources. Prior work by co-authors in this study and others have attempted to quantify the effect of mobility on transmission of SARS-CoV-2 in Seattle, WA during a similar time period. They find that pathogen circulation dropped substantially after the initiation of stay-at-home orders in March 2020. During this period, mobility was a positive, leading indicator of transmission of all endemic viruses and lagged SARS-CoV-2 activity. Mobility was briefly predictive of SARS-CoV-2 transmission when restrictions 30 relaxed in summer 2020 but associations weakened in subsequent waves (1)

1. Perofsky AC, Hansen C, Burstein R, Boyle S, Prentice R, Marshall C, et al. Human mobility impacts the transmission of common respiratory viruses: A modeling study of the Seattle metropolitan area [Internet]. medRxiv; 2023 [cited 2024 Feb 1]. p. 2023.10.31.23297868. Available from: https://www.medrxiv.org/content/10.1101/2023.10.31.23297868v1

From a qualitative perspective, looking at Figure 5A, we can see that the periods with the most persistent transmission chains align with the arrival of a new variant with a transmissibility advantage (Alpha and Delta) and the lifting of stay at home orders, with South King County consistently experiencing more persistent transmission chains than North. We see increases both in the summer and the winter suggesting a lack of correlation with climatic factors and more with the major waves of COVID cases. To adequately answer this question, an independent, carefully-designed study employing longitudinal data analysis methods such as logistic regression with random effects or generalized estimating equations

We have integrated this information into the Results section as follows (new information in *italics*):

We next analyzed the posterior set of phylogenies produced by the MASCOT-GLM analysis to understand within and between region viral circulation. Given the higher estimated *Ne* in South King County, we quantified the average persistence time of viral

transmission chains in each region (Fig 5A, see Methods). While the average monthly persistence time remained relatively equal between the two regions during the early stages of the epidemic, following May 2020 up until 2022, we see that transmission chains in South King County consistently have significantly higher persistence times than in North King County, with the mean local transmission length averaged over the entire time period of 21.5 days in South King County and 13.5 days in North King County. *We see an increase in average persistence times in both regions during large waves of COVID-19 cases attributable to the introduction of a new variant with a transmissibility advantage (such as in late 2020- early 2021 with the introduction of Alpha) and the relaxation of stay-at-home order, with South King County consistently having longer persistence times.*

Part III – Minor Issues: Editorial and Data Presentation Modifications
Please use this section for editorial suggestions as well as relatively minor modifications of existing data that would enhance clarity.

Reviewer #1: - The statement “Figure 2c shows all clusters greater than size five with respective posterior support for inferred ancestral states.” is redutant as it repeats the earlier statement.
Agreed. We have removed this statement.
- Please label control measures in Fig. 1 C and D, and Fig. 5A
Both figures have been adjusted accordingly.

Previous Fig 1 (now Fig 2):

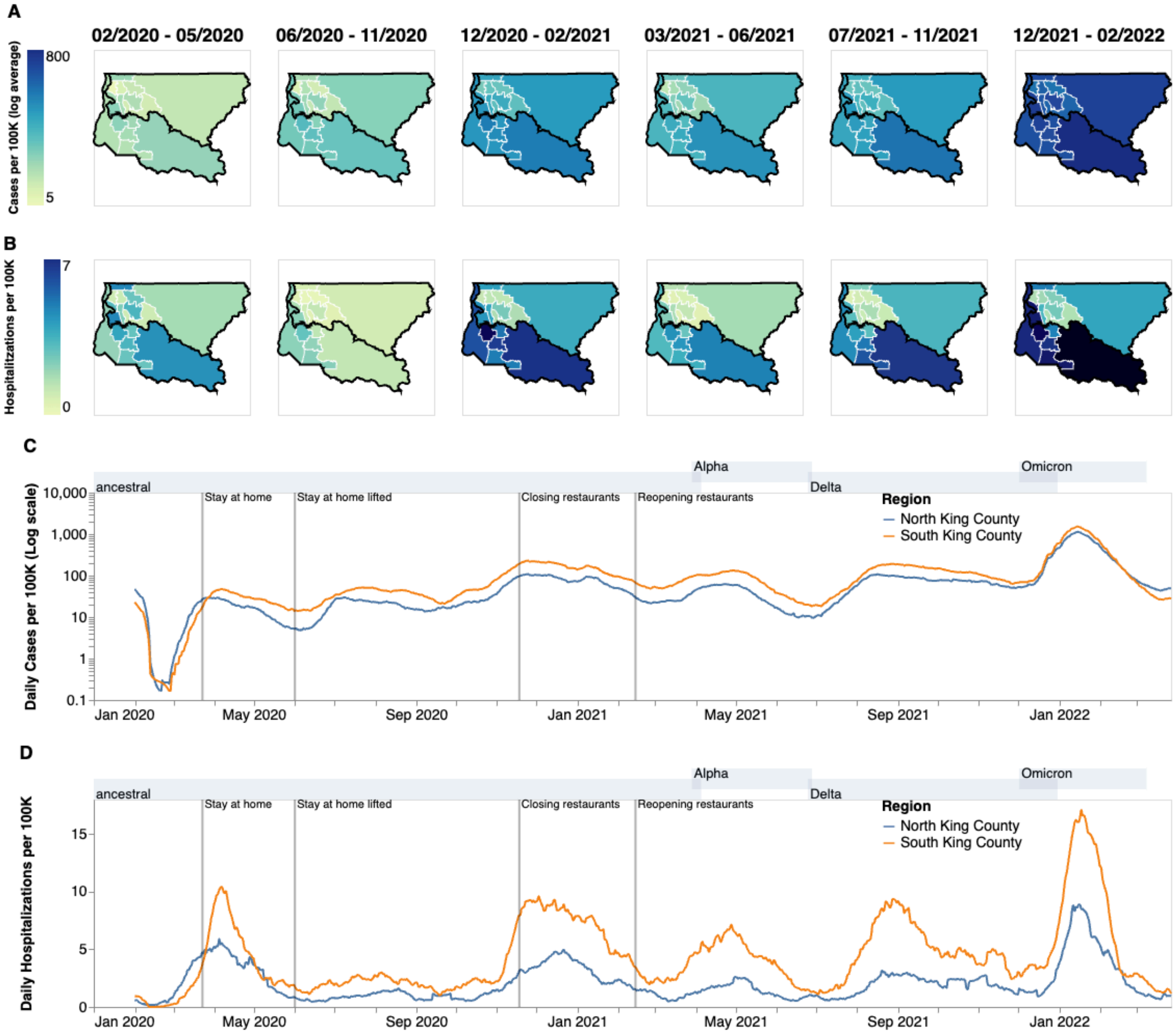

Previous Fig 5 (now Fig 6)

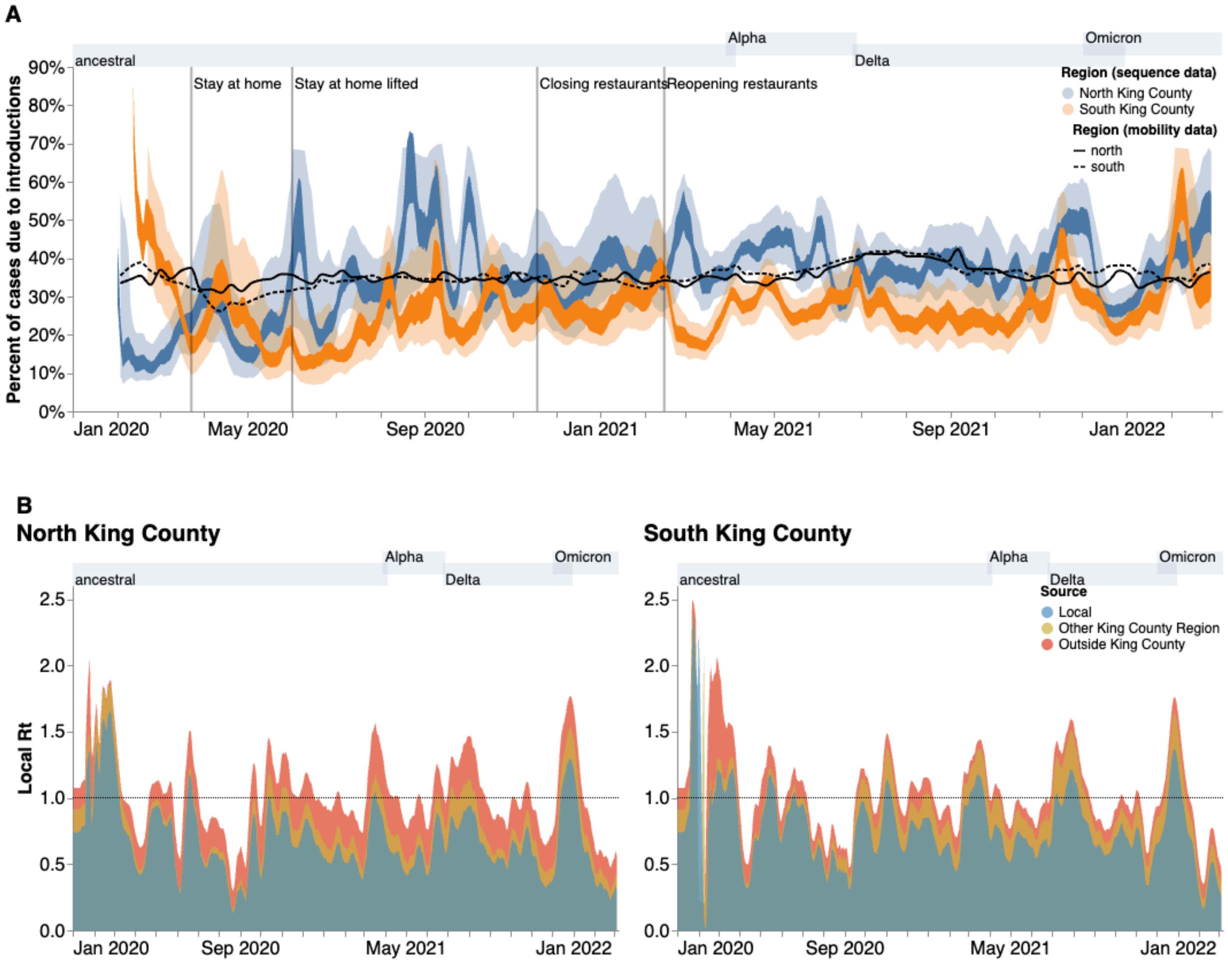

- Typo? mzy -> myz

It is not completely clear as to where the reviewer is referring to. As such, we have gone through the manuscript again and checked the correct labeling of each migration rate variable in our equations.

The order of the subscripts refers to the direction of migration and changes if we are looking forward-in-time or backwards-in-time. If we are interested in the percentage of cases due to introductions π for region *y*, then we use either m^f_{yz} to denote the forward migration rate from region *y* into region *z* or m^b_{zy} to denote the backwards migration rate from region *z* into region *y*. In order to get the number of introductions into region *y* using backwards migration rates (which MASCOT-GLM uses), we need to multiply the backwards migration rate m^b_{zy} with the viral population at region *z*, Ne_z, as we are interested in the *number* of exports from *z* into *y*. To make

this a bit clearer, we have expanded the explanation a bit in the Methods as follows (new changes in *italics):*

> We estimated the number of new local cases at time *t* by assuming the local epidemic in each King County region follows a simple transmission model, in which we estimate the number of new cases at time *t* as the product of the transmission rate β (new infections per day per individual) multiplied by the number of people already infected in that region *I*. For the number of introductions, we similarly assumed that the number of introductions equals the product of the rate of introduction (introductions per day, which we refer to as migration rate *m*) and the number of people already infected in that region *I. We use the number of infected individuals in the destination region rather than the origin region for calculating the number of introductions since the approximate structured coalescent approach models epidemic processes as backwards-in-time, resulting in the equation containing only information about the number of infected individuals in the destination region.*

- In the sentence "21,976 genome sequences from around the world downloaded from GISAID", the contextual sequences for Omicron, Delta, Alpha and other lineages exceed this total number.

Thank you for catching this; it reflected a previous iteration of the analysis. We have adjusted the numbers accordingly.

Reviewer #2: 1. What is most interesting about this study is the comparison of transmission dynamics between North King County and South King County and how their differ. (North is more driven by outside introductions, whereas South has more community transmission). But while the authors may be familiar with the differing demographics of these two regions, the reader has to make it all the way to Figure 6 (page 22) to get a picture of how these two regions differ socioeconomically, in % essential workers, etc. It would be helpful to move Figure 6 to the beginning of the paper and include some text in the introduction. Can the authors provide any clarity on why there seems to be such a stark socioeconomic line in the middle of King County? Why do adjacent zip codes in the middle of the county differ so much in socioeconomic measures? Could you also provide a map with population density in Figure 6? Possibly vaccination rates?

Thank you for this suggestion. We agree and have moved Figure 6 and the associated discussion paragraph about the correlation between difference in case counts and socioeconomic inequities into the introduction and have renamed it Figure 1 in order to provide readers with more context at the beginning of the article. We have also added in a map with population size as new figure 1E.

Additionally, we have added more context into the Discussion regarding historical redlining and racial segregation in Seattle that has promoted unequal health outcomes even in adjacent zipcodes in King County : (new additions in *italics*)

> Given the smaller population size in South King County, one potential explanation for higher local spread is reduced access to social and economic capital and health care resources needed to curb community transmission. Previous studies looking at SARS-CoV-2 test positivity in King County at a census tract level have found that a higher test positivity was associated with various socioeconomic indicators including lower educational attainment, higher rates of poverty, and high transportation costs (15,16). Additionally, they found that communities with a higher proportion of people of color, which are more likely to be located in South King County, were also associated with higher test positivity in 2020. Hansen et al. (16), specifically found that having a place of residence in South King County was associated with SARS-CoV-2 test positivity. *The stark contrast in health outcomes between North and South King County has been previously attributed to historical redlining and systemic racism, whereby decades of racial segregation prevented communities of color from residing in northern areas of Seattle and were forced into the south into present day South King County (17,18).*

Relevant references:

17.

Seattle's Race and Segregation Story in Maps 1920-2020 - Seattle Civil Rights and Labor History Project [Internet]. [cited 2024 Jan 31]. Available from: https://depts.washington.edu/civilr/segregation_maps.htm

18.

Bramble K, Blanco MN, Doubleday A, Gassett AJ, Hajat A, Marshall JD, et al. Exposure Disparities by Income, Race and Ethnicity, and Historic Redlining Grade in the Greater Seattle Area for Ultrafine Particles and Other Air Pollutants. Environmental Health Perspectives. 2023 Jul;131(7):077004.

2. This study examines transmission in the context of non-pharmaceutical interventions like stay at home orders and closing bars. What about closing schools?

Washington State closed down public schools on March 16th, 2020, which was only a week before the statewide shelter in place was issued on March 23rd, 2020. Similarly, public schools returned to in-person instruction on April 5th, 2021, which is near to the date of restaurant reopening at the end of February 2021. Given the significant overlap in opening and closing

dates with the NPIs already examined in the manuscript, we chose not to include them in our figures for the sake of clarity.

We have added in this context in to the methods section under "Empirical Predictors" (new information in *italics)*:

> We also used dates of non-pharmaceutical interventions (NPIs) in WA and between-region mobility to inform migration rates between North and South King County. Dates of NPIs were found as part of the COVID-19 US State Policy Database (37). NPIs included are start and end of emergency stay at home orders as well as closing and reopening of bars and restaurants. *We chose not to include the opening and closing of public schools due to a high degree of overlap with the NPIs already included. Washington State closed down public schools on March 16th, 2020, which was only a week before the statewide shelter in place was issued on March 23rd, 2020. Similarly, public schools returned to in-person instruction on April 5th, 2021, which is near to the date of restaurant reopening at the end of February 2021.*

3. I could not find any MCC trees or ML trees in the GitHub repository. Figure 2 is missing background data so the King County data is more visible but there are some surprisingly large King County clusters, given how fluidly SARS-CoV-2 mixes within and between states. Are these monophyletic? And the very long branches (appears to be delta? some labeling of the different variants in the tree would help), are these chronically infected people? One of the branches spans >6 months. What % of transmission chains (maybe with at least 10 sequences) are only found in one region (north or south) versus both?

The ML trees can be found at https://github.com/blab/ncov-king-county/tree/main/nextstrain_build/auspice . Additionally, we have added in a new supplementary figure 1 (reproduced below) to not only show the data underlying our analyses but also to highlight the range of viral genetic diversity found within King County WA throughout the time period studied. In the figure legend, you'll also find links to nextstrain.org where you can explore the trees interactively.

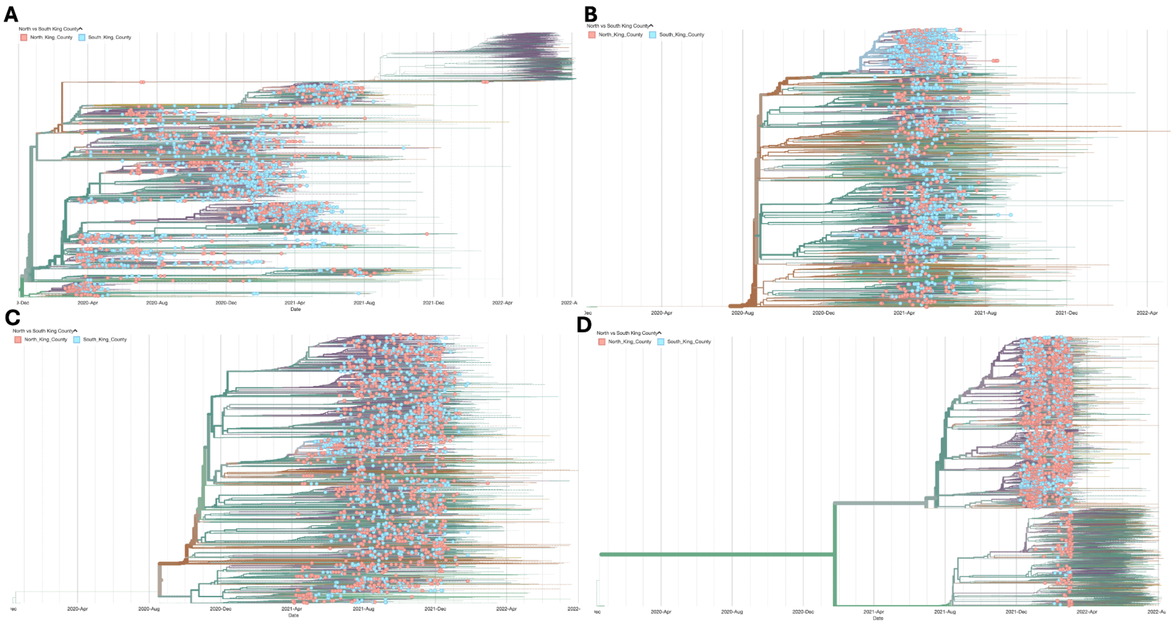

Supplementary Figure 1: Time-resolved maximum likelihood phylogenies for King County, WA by dominant variant wave with sample collection dates between February 1 2020 and March 6 2022. Trees are filtered to highlight genomes from King County among contextual sequences from around the globe. Tip color represents the region within King County, with pink corresponding to North King County and blue representing South King County. Branches are colored based on inferred ancestry. Panel **A** represents all variant clades excluding Alpha, Delta, and Omicron (the full tree can be explored interactively at https://nextstrain.org/groups/blab/ncov-king-county/other), the other panels represent Alpha (**B**, https://nextstrain.org/groups/blab/ncov-king-county/alpha), Delta (**C**, https://nextstrain.org/groups/blab/ncov-king-county/delta), and Omicron (**D**, https://nextstrain.org/groups/blab/ncov-king-county/omicron).

The MCC trees were not uploaded in GitHub previously due to an error in .gitignore. They can now be found in https://github.com/blab/ncov-king-county/tree/main/mascot_glm/results/.

Previous Figure 2 (now Figure 3) does not include background data as background sequences were mainly used for the identification of King County clusters. These clusters were then subsampled and only the King County sequences were included in the MASCOT-GLM analysis, which is what is displayed in the figure. To make this clearer, we have added in the following into the Methods under the subheading *MASCOT GLM on multiple local outbreak clusters:*

> To analyze the transmission dynamics within and between South and North King County, we used an adapted version of MASCOT (28) on the 3000 subsampled King County clusters and sequences.

Given that we do not have access to individual clinical information, we are unable to assess the immune status of each infected individual to see if some are chronically infected.

While some King County clusters in our dataset were found to be monophyletic, it is possible that some of the larger clusters, and subsequently some of the very long branches, are a consequence of very rapid transmission and sparse sampling that leads to a polytomy in the ML tree.

We looked at the sequence with the very long transmission time found in Figure 3 via our Delta phylogeny. As seen in the screenshot below, where the tree is filtered to highlight the cluster and sample in question, we can see that this sequence is part of the larger cluster of which the other cluster members are a part of. This is a long branch off of the base of this cluster. It has the expected number of mutations for a branch of this length of time. Thus, we expect this represents a circulating transmission chain that happened to not have intermediate samples taken from it. Our approach infers the parsimonious conclusion that this transmission chain was circulating locally in King County until this WA-CDC-UW21121797659/2021 sample was taken. This cluster can be explored interactively at https://nextstrain.org/groups/blab/ncov-king-county/delta?c=cluster&d=tree&f_cluster=905&m=div&p=full

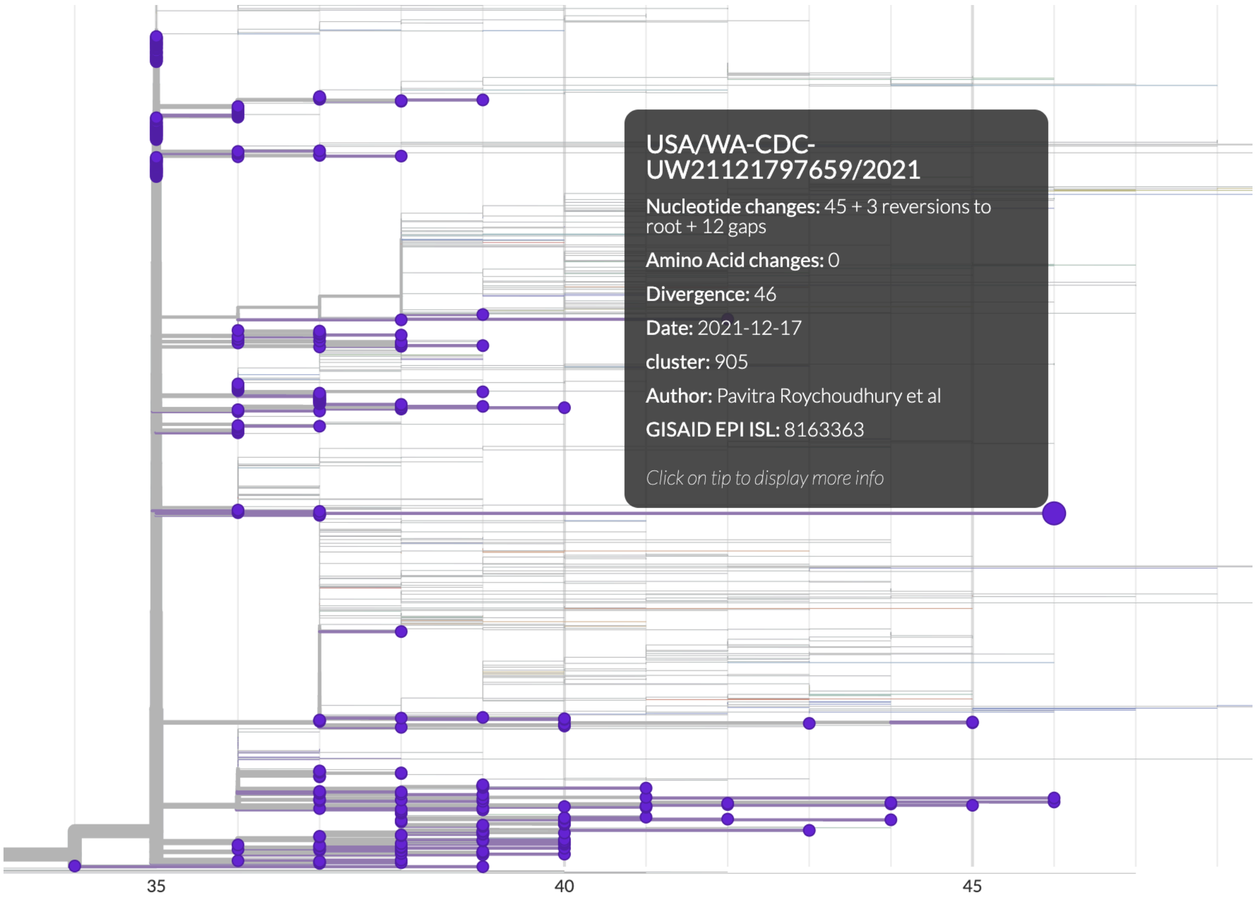

This effect has been previously well documented (1) and the use of GLM approaches, like MASCOT-GLM which we use here, have been previously applied to ameliorate this issue (2).

1. Morel B, Barbera P, Czech L, Bettisworth B, Hübner L, Lutteropp S, et al. Phylogenetic Analysis of SARS-CoV-2 Data Is Difficult. Molecular Biology and Evolution. 2021 May 1;38(5):1777–91.
2. Lemey P, Ruktanonchai N, Hong SL, Colizza V, Poletto C, Van den Broeck F, et al. Untangling introductions and persistence in COVID-19 resurgence in Europe. Nature. 2021 Jul;595(7869):713–7.

We have added a bit more context to the limitations section of the Discussion (new additions in *italics)*

> Our phylodynamic analyses are conditioned on inferred King County sequence clusters that are found through the incorporation of contextual sequences from around the world into a temporally-resolved phylogeny. As such, it is possible that differential sampling from other locations could impact our identified clusters. *Limited SARS-CoV-2 sequence diversity, especially during periods of rapid transmission, could impact our ability to break up larger clusters (23), which might lead to collapsing multiple introductions into King County into shared clusters. Prior studies have used GLM approaches to ameliorate this bias (24), similar to our use of MASCOT-GLM.* Optimally, we would like to avoid having to a priori define local outbreak clusters entirely by, for example, explicitly accounting for locations outside of King County in the model. This is currently not possible due to the additional computational cost of explicitly considering an outside deme. Additionally, Bayesian coalescent models assume random sampling of infected individuals, meaning that targeted sampling, such as super spreader events or contact tracing, could bias our phylodynamic estimations. Such sampling from outbreak analyses may also not be constant through time, complicating *Ne* inferences. Lastly, our *Rt* calculations assume that the change in *Ne* over time is proportional to the change in the number of infected individuals over time.

We also checked to see what percentage of transmission chains with at least 10 sequences were only found in one region vs both. Out of the 90 clusters, only 4 of those were found only in one region. Of those four, three were found only in South King County.

4. In your models, did you use the same parameters throughout, or change them as new variants arrived?

The same predictors for MASCOT-GLM were used throughout the entire time period. We used hospitalizations, cases, and percent of the population with COVID-19 association immunity to inform the effective population size estimates and mobility and NPI dates to inform the migration rates. We have included the estimated coefficients of each predictor in new Figure 4. Predictors, however, are time-varying, accounting for differences as new variants are introduced.

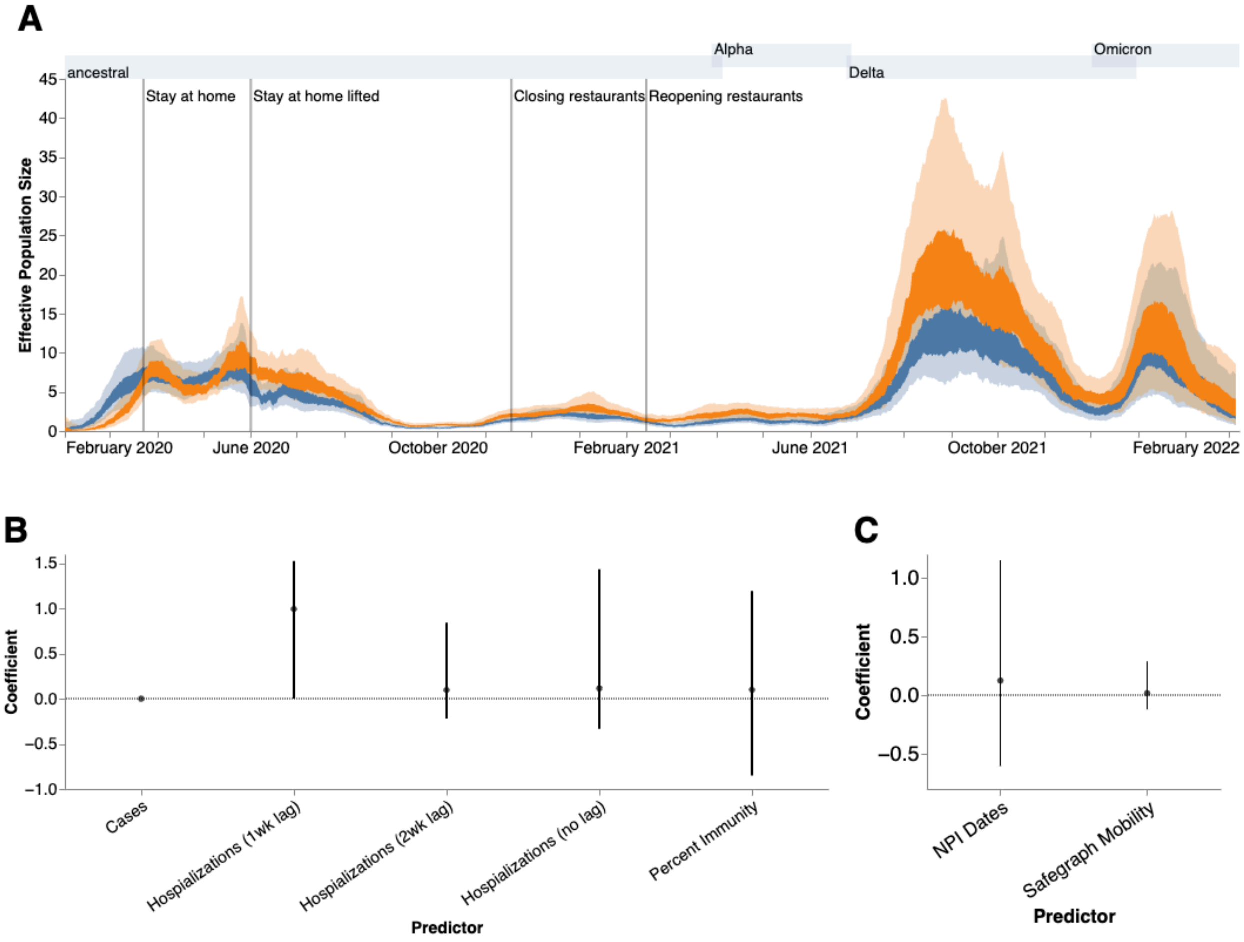

5. It would be helpful in all the figures to label the variants in the different waves (e.g., Fig 3). Most readers probably know them, but when people this read this paper 5-10 years from now they may forget.

Thank you for your suggestion. We have updated each figure with bars and labels over the figures to represent the different variants, as seen in the example below:

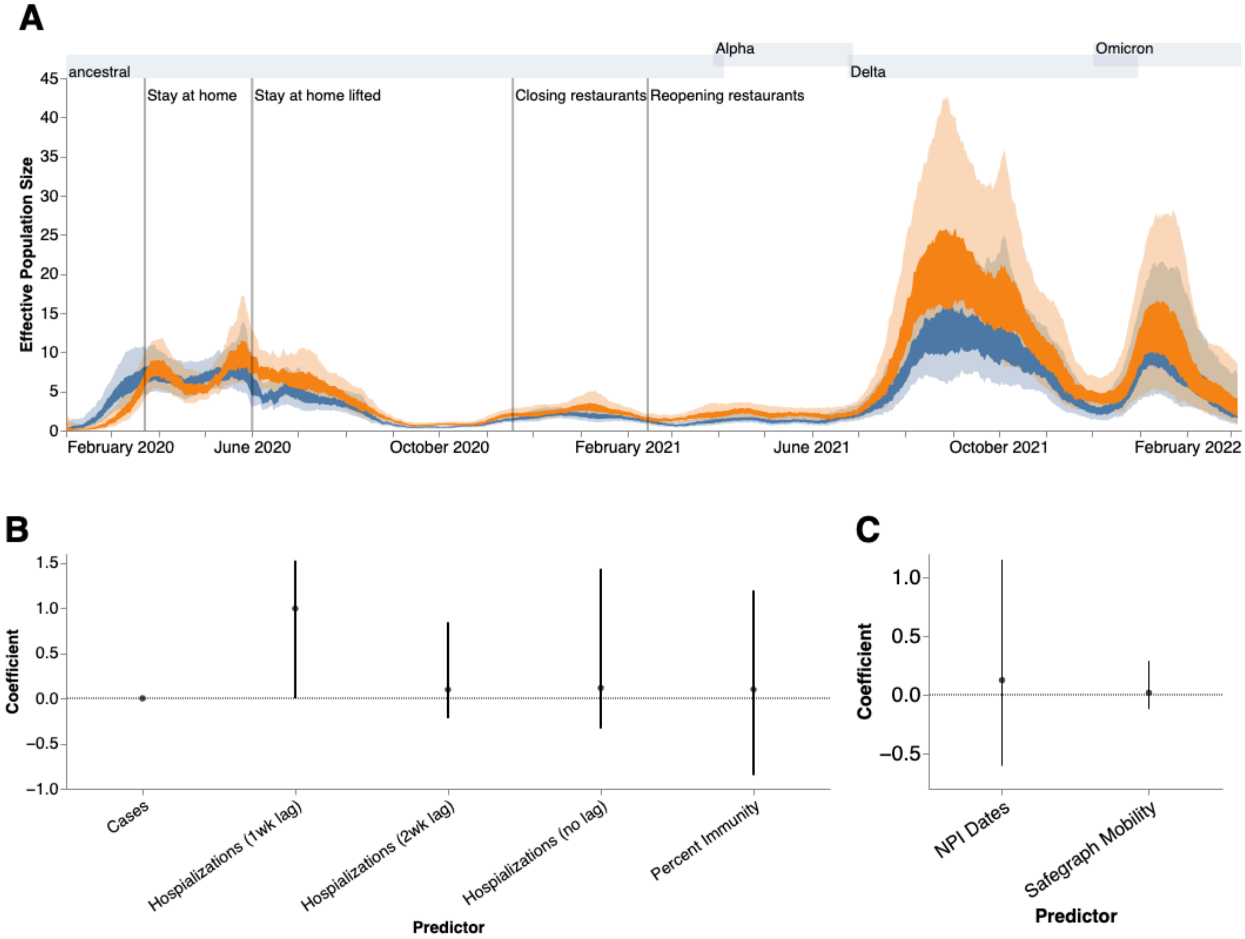

6. Could your discussion include some more concrete examples of how this kind of molecular epidemiology could, in retrospect, change how policy makers acted during the pandemic? Based on these findings, could they have targeted interventions differently or more strategically? Is one takeaway that a region's COVID control is only as good as its weakest link, because any pocket where cases are high will quickly seed other areas? What is your main takeaway?

We agree that more concrete examples would help highlight the utility of our approach. As such, we have updated the final paragraph of the Discussion with examples on how our results could promote more targeted public health efforts (newly added text in *italics*):

> The transmission dynamics of the SARS-CoV-2 pandemic have been highly heterogeneous across countries. Here we show that even different areas of the same metropolitan region can have different trajectories. *Changes in incidence throughout the course of an epidemic can be driven by changes in local transmission, importations, or*

both. Common methods to estimate incidence and changes in incidence via Rt often ignore or are unable to quantify these differences (25–27), leading to situations where local health departments have limited information with which to tackle growing case counts. Our local scale genomic epidemiology approach can reveal these differences by quantifying the contribution of importations and local transmission on Rt (Fig 6B) through the joint integration of genomic and epidemiological information. Quantifying changes and differences in contribution to incidence can directly lead to tailored interventions. For example, in an area where incidence is driven mostly by outside viral introductions, interventions could focus on limiting their impact by implementing testing at the airport or quarantine for recent travelers. Meanwhile, ramping up testing, vaccination, and masking as well as providing medical and economic aid to promote quarantine and isolation without furthering income inequities could be more impactful for areas where local community transmission is the main driver of epidemic growth.

PLOS authors have the option to publish the peer review history of their article (what does this mean?). If published, this will include your full peer review and any attached files.

If you choose "no", your identity will remain anonymous but your review may still be made public.

Do you want your identity to be public for this peer review? For information about this choice, including consent withdrawal, please see our Privacy Policy.

Reviewer #1: No

Reviewer #2: No

Figure Files:

While revising your submission, please upload your figure files to the Preflight Analysis and Conversion Engine (PACE) digital diagnostic tool, https://pacev2.apexcovantage.com. PACE helps ensure that figures meet PLOS requirements. To use PACE, you must first register as a

user. Then, login and navigate to the UPLOAD tab, where you will find detailed instructions on how to use the tool. If you encounter any issues or have any questions when using PACE, please email us at figures@plos.org.

Data Requirements:

Please note that, as a condition of publication, PLOS' data policy requires that you make available all data used to draw the conclusions outlined in your manuscript. Data must be deposited in an appropriate repository, included within the body of the manuscript, or uploaded as supporting information. This includes all numerical values that were used to generate graphs, histograms etc.. For an example see here: http://www.plosbiology.org/article/info%3Adoi%2F10.1371%2Fjournal.pbio.1001908#s5.

Reproducibility:

To enhance the reproducibility of your results, we recommend that you deposit your laboratory protocols in protocols.io, where a protocol can be assigned its own identifier (DOI) such that it can be cited independently in the future. Additionally, PLOS ONE offers an option to publish peer-reviewed clinical study protocols. Read more information on sharing protocols at https://plos.org/protocols?utm_medium=editorial-email&utm_source=authorletters&utm_campaign=protocols

References:

Please review your reference list to ensure that it is complete and correct. If you have cited papers that have been retracted, please include the rationale for doing so in the manuscript text, or remove these references and replace them with relevant current references. Any changes to the reference list should be mentioned in the rebuttal letter that accompanies your revised manuscript. If you need to cite a retracted article, indicate the article's retracted status in the References list and also include a citation and full reference for the retraction notice.
